# Endothelial progenitor cell derived exosomes mediated miR-182-5p delivery accelerate diabetic wound healing via down-regulating PPARG

**DOI:** 10.7150/ijms.78790

**Published:** 2023-02-13

**Authors:** Peng Li, Guanhao Hong, Weiqiang Zhan, Mingzhu Deng, Chenlin Tu, Jinsong Wei, Hao Lin

**Affiliations:** 1 Stem Cell Research and Cellular Therapy Center, Affiliated Hospital of Guangdong Medical University, Zhanjiang 524001, China; 2 Orthopedic Center, Affiliated Hospital of Guangdong Medical University, Zhanjiang, 524001, China

**Keywords:** Endothelial progenitor cell derived exosomes, diabetic wound healing, miR-182-5p, PPARG Endothelial progenitor cell derived exosomes, diabetic wound healing, miR-182-5p, PPARG

## Abstract

Diabetic wound is one of the most common and serious complications of diabetes, which is characterized by abnormal number and quality of wound repair related cells. Previous studies have shown that human endothelial progenitor cells derived exosomes (EPCs-EXO) can promote diabetic wound healing through modulating vascular endothelial cell function. The purpose of this study was to investigate the biological effects and molecular mechanisms of EPCs-EXO on diabetic wound healing. The regulation of EPCs-EXO on human immortalized epidermal cell line HaCaT in high glucose (HG) environment was evaluated. Our data showed that EPCs-EXO promoted the proliferation, migration, while inhibited apoptosis of HaCaTs challenged by HG via elevating miR-182-5p expression level* in vitro*. Skin wound healing was significantly enhanced by EPCs-EXO in diabetic mice. Moreover, bioinformatics analyses and luciferase reporter assay indicated that exosomal miR-182-5p was bound to PPARG 3' UTR sequence and inhibited the expression of PPARG. Collectively, our findings provided a new role of EPCs-EXO in the clinical treatment of diabetic skin wounds.

Diabetic wound is one of the most common and serious complications of diabetes, which is characterized by abnormal number and quality of wound repair related cells. Previous studies have shown that human endothelial progenitor cells derived exosomes (EPCs-EXO) can promote diabetic wound healing through modulating vascular endothelial cell function. The purpose of this study was to investigate the biological effects and molecular mechanisms of EPCs-EXO on diabetic wound healing. The regulation of EPCs-EXO on human immortalized epidermal cell line HaCaT in high glucose (HG) environment was evaluated. Our data showed that EPCs-EXO promoted the proliferation, migration, while inhibited apoptosis of HaCaTs challenged by HG via elevating miR-182-5p expression level* in vitro*. Skin wound healing was significantly enhanced by EPCs-EXO in diabetic mice. Moreover, bioinformatics analyses and luciferase reporter assay indicated that exosomal miR-182-5p was bound to PPARG 3' UTR sequence and inhibited the expression of PPARG. Collectively, our findings provided a new role of EPCs-EXO in the clinical treatment of diabetic skin wounds.

## Introduction

Diabetes is a kind of chronic metabolic disease that has increases in prevalence over time and poses a serious economic burden both in developing and developed countries 1. Impaired would healing damages is one of the most common complications of diabetes and the barrier function of skin tissues increases the chance of infection, leading to necrosis or even amputation 2. In addition, the wound healing has increased the cause over the past years and have a significant impact on patients' physical and psychological health. Diabetic wounds are caused by the dysfunction of angiogenesis and the proliferation, differentiation and migration of endothelial cells 3. Previous studies have indicated that the application of stem cells derived exosomes can promote the repair of diabetic wounds through signal transduction 4-6.

Exosomes are extracellular vesicles with diameters between 30-200 nm which were widely found in the supernatant of cell culture and various body fluids 7. Exosomes contain a large amount of genetic information and abundant RNA types, including mRNA, miRNA, or lncRNA 8. The ability of exosomes to transport specific DNA or RNA to target cells are used for gene therapy to regulate the bioactivity of target cells 9. Most cells secrete exosomes and participates in variable biological processes 10. Previous studies have shown that exosomes can promote tissue repair and regeneration in various animal models, such as cardiac muscle deficiency 11, pulmonary fibrosis 12, pulmonary edema 13, kidney injury 14, diabetes 15, and fracture healing 16.

Endothelial progenitor cells (EPCs) are the precursor cells of vascular endothelial cells. Under the stimulation of physiological or pathological factors, EPCs can mobilize from bone marrow to peripheral blood to participate in the repair of damaged blood vessels 17. Studies have shown that EPCs play an important role in cardiovascular and cerebrovascular diseases, peripheral vascular diseases, tumor angiogenesis and wound healing via their differentiation and proliferation 18, 19. However, the adhesion, migration, proliferation and *in vitro* angiogenesis of EPCs were significantly decreased under HG concentration with a time-dependent manner 20, 21. It has been found that exosomes secreted by EPCs regulating the functions of endothelial cells and have beneficial effects on microvascular repair 22. However, the mechanisms of the exosomes secreted by EPCs in diabetes impaired would remain unclear.

MicroRNAs (miRNAs) with a length of about 22nt are a class of evolutionarily highly conserved small non-coding RNAs, inhibit post-transcriptional gene expression and play an important role in regulating genes expression. miRNAs in exosomes derived from EPCs play a role in promoting angiogenesis and promote tissue regeneration and repair 23, 24. Exosomes derived from ADSCs transport some functional miRNAs to target cells to activate endogenous repair machinery, such as miRNA-126, miRNA-130a and miRNA-132^25^-^27^. Exosomes derived from BMSCs carrying miRNA-125b to protect myocardial cells from reperfusion injury 28. Exosomes derived from MSCs carrying miRNA-126-3p showed significant activation of PI3K/AKT and MAPK/ERK1/2 pathways, and showed dose-dependent promoting proliferation of dermal fibroblasts and human dermal microvascular endothelial cells 29. Current research evidence suggests that the treatment of exosomes derived from stem cells is an effective way to treat diabetic wounds.

In this study, we investigated the therapeutic efficacy of EPCs-EXO in diabetes cutaneous wound healing. HaCaTs with EPCs-EXO were treated under HG condition, the proliferation and migration were promoted, while the apoptosis was inhibited of HaCaTs via elevating miR-182-5p expression level and inhibiting PPARG expression. Moreover, skin wound healing was significantly enhanced by EPCs-EXO in diabetic mice. Our results indicting the significance role of EPCs derived exosome in treating diabetic cutaneous wounds.

## Methods and materials

### Ethics statement

Umbilical cord blood was obtained from the Affiliate Hospital of Guangdong Medical University and the collection was approved by the hospital ethics committee and informed consent of the mother was obtained (PJ2019-017KT). All animal experiments were performed in accordance with the guidelines of the Animal Care and Use Committee of the Affiliate Hospital of Guangdong Medical University (GDY1902014).

### Cell culture

Human immortalized epidermal cell line HaCaT was obtained from the American Type Culture Collection (ATCC, Manassas, VA, USA) and cultured in Dulbecco's Modified Eagle Medium (DMEM) supplemented with 10% fetal bovine serum (FBS) and 1% penicillin/streptomycin at 37 °C in a humidified atmosphere with 5% CO_2_. HaCaT cells were seeded in 6-well plates and incubated in media containing normal glucose (6 mM glucose) or high glucose (30 mM glucose).

### Isolation and characterization of EPCs

The layer of peripheral blood mononuclear cells was isolated via a Ficoll-hypaque density gradient centrifugation from the human cord blood samples and then re‑suspended in EBM-2 basal medium. After cells had adhered, unattached cells were removed and the medium was changed every 2 days for 1 week and the EPCs were obtained and identified with immunofluorescence staining. EPCs were stained with FITC-conjugated VEGFR2, FITC-conjugated CD34 and PE-conjugated CD133 antibodies at 4 °C for 30 min. Nuclei were stained with DAPI at room temperature for 10 min. Micrographs were acquired by fluorescence microscopy and cells with double‑positive fluorescence were considered to be EPCs. For further identification of the EPCs, cells were incubated with Dil‑Ac‑LDL for 4 h at room temperature and fixed by 4% paraformaldehyde for 20 min, followed by incubation with FITC‑UEA‑1 for another 1 h. Then DAPI was incubated for 10 min at room temperature. Then cells were examined with fluorescence microscopy.

### Isolation and identification of EPCs-EXO

The third generation of EPCs were taken and the culture medium of supernatant was collected after 48 h. The separation of EPCs-EXO was used with density gradient centrifugation as previous studies mentioned. Briefly, supernatant was centrifuged at 300 g for 10 min to remove cells, centrifuged at 2000 g for 10 min to remove the dead cells, and then centrifuged at 10,000 for 60 min to remove the debris and centrifuged at 100,000 for 60 min to obtain the EPCs-EXO. The EPCs-EXO were observed by transmission electron microscope and the membrane surface marker proteins CD63, TSG101 and HSP70 were analyzed by Western blot. Particle size distribution of the EPCs-EXO was detected by nanoparticle tracking analyzer.

### Uptake of EPCs-EXO by HaCaT cells

PKH26 and PKH27 (1:1 mix, 2 × 10-6 M, Sigma-Aldrich) were added into EPCs-EXO suspension for 15 min and washed with PBS and then centrifuged at 100,000 g for 60 min. Then PKH26 and PKH27 labeled EPCs-EXO were co-cultured with HaCaT cells for 24 h. Uptake of exosomes was observed under a fluorescence microscopy.

### Sequencing and bioinformatics analysis of miRNAs

Total RNA from EPCs-EXO was isolated using miRNeasy Serum/Plasma Kit (QIAGEN) according to the protocol. Library preparation for small RNA Sequencing was prepared with using VAHTSTM Small RNA Library Prep Kit for Illumina® (Vazyme Biotech) and sequenced by HiSeq 2500 (Illumina, USA) at Aksmics (Shanghai, China). In addition, total RNA from HaCaT cells treated with EPCs-EXO or not under HG condition were collected for microarray analysis. Both sequencing and microarray analysis of differentially expressed miRNAs were then identified according to Fold change and P-value. Fold change >2 or <-2 and a p value <0.05 were set as the threshold for the up-and downregulated genes. The heatmap package in R (version 3.1.3) was used to construct the differential genes expression heatmap and the regulating target genes were predicted using the miRDB database (http://mirdb.org) and the intersection between these predictions was performed by protein-protein interaction (PPI) network.

### Luciferase reporter assay

Luciferase reporter assay was performed to explore the potential target gene of miR-182-5p. The 3'-UTR sequence of PPARG wild type (PPARG-WT) and PPARG mutant (PPARG-mut) were cloned into pmirGLO dual-luciferase miRNA target expression vector. Luciferase reporter plasmids and miR-182-5p mimics or mimic controls were co-transfected in HEK293T cells. The relative luciferase activity was detected by the Dual-Luciferase Reporter System (Promega, Madison, WI, USA) after 48 h transfection.

### Cell viability assay

The cell proliferation was utilized with the Cell Counting Kit 8 (CCK8) assay. HaCaTs were seeded in 96-well plates with 100 μL of medium per well. Cells were treated with normal glucose, high glucose (HG), EPCs-EXO and high glucose incubated with EPCs-EXO for 48 h. Then 10 μL CCK8 reagent was added to each well and incubated for 1 h at 37 °C in a humidified atmosphere with 5% CO_2_. Then the absorbance was measured at a wavelength of 450 nm.

### Cell adhesion assay

HaCaTs were seeded in 96-well plates treated with normal glucose, HG, EPCs-EXO and HG plus EPCs-EXO for 4 h. Then these cells were incubated with MTT for 90 min and non-adhered cells were washed out with PBS. The adhered cells were lysed with dimethylsulfoxide (DMSO) and quantitated by measuring absorbance at 540 nm.

In addition, ELISA was also used to measure the expression of extracellular adhesion matrix protein Col-IV and HA in EPCs-EXO co-cultures in NG/HG condition. Briefly, 96-well plate was pre-coated with Col-IV and HA monoclonal antibody at 4°C overnight and washed with PBS. HaCaTs were treated with normal glucose, HG, EPCs-EXO and HG plus EPCs-EXO for 48 h and the supernatant was collected and added into the antibody‐coated plate and incubated for 1 h at room temperature. After washing the plate, the enzyme substrate was added and incubated at room temperature for 15 min. The reaction was stopped and absorbance at 500 nm was measured to calculate the medium concentration of COL IV and HA.

### 2.10 Wound scratch assay

HaCaTs were seeded in a 6-well plate and scraped with a sterile 200 μL tip for 3 lines and washed twice with PBS to remove the cell fragmentation. Then cells were incubated with or without EPCs-EXO under HG medium containing 1% fetal bovine serum for 0, 24, 48 and 72 h. The cells were photographed under an inverted microscope (Olympus, Tokyo, Japan) and the distances between the wound edges were measured using Image J software.

### Apoptosis assay

The apoptosis of HaCaTs from normal glucose, HG, EPCs-EXO and HG plus EPCs-EXO groups were evaluated using Annexin V/PI flow cytomety staining. Cells were digested with trypsin (without EDTA) and washed with PBS and then suspended with 100 μL binding buffer containing 5 μL of Annexin V and 10 μL PI for 5 min at room temperature. Then added 300 μL binding buffer and analyzed by a BD Flow Cytometer.

### Cell cycle assay

HaCaTs were treated with normal glucose, HG, EPCs-EXO and HG plus EPCs-EXO for 48 h. Cells were digested with trypsin and washed with PBS and then fixed with 70% ethanol overnight. After washing with PBS, the cells were stained with PI (50 μg/mL) and RNase A (100 μg/mL) for 30 min at room temperature. Then added 500 μL PBS and analyzed by a BD Flow Cytometer.

### Colony formation assay

HaCaTs were incubated with normal glucose, HG, EPCs-EXO and HG plus EPCs-EXO for 48 h. Then cells were digested with trypsin and seeded at a density of 300 cells per well in six-well plates for 2 weeks in full medium. Finally, colonies were stained with 1% crystal violet for 10 minutes at room temperature, and the number of the colonies was counted under a microscopy.

### RNA isolation and qRT-PCR analysis

Total RNA of HaCaTs treated with HG or HG plus EPCs-EXO for 48 h were carried out by TRIzol® reagent (Thermo Fisher Scientific, USA) and the reverse transcription of purified RNA converted into cDNA using TransScript® One-Step gDNA Removal and cDNA Synthesis SuperMix kit (Beijing, China) and qRCR was performed using TransScript® Green One-Step qRT-PCR SuperMix (Beijing, China) and a Bio-Rad qPCR system (CFX96, USA). The relative miRNA expression levels were normalized to the internal control GAPDH.

### Western blotting

Total proteins were extracted from HaCaTs using RIPA lysis buffer with proteinase inhibitor and 20 μg protein was separated by 10%-12% SDS-PAGE, then transferred into the PVDF membrane and blocked at room temperature for 2 h with 5% bovine serum albumin. Membranes were incubated with primary antibody at 4°C overnight and secondary antibodies for 1 h at room temperature and visualized using a chemiluminescent detection system. Primary antibodies were used in the Western blotting analysis: anti-CD63, anti-TSG101, anti-HSP70, anti-PPARG, anti-MMP1, anti-CTNNB, anti-FN1, and anti-GAPDH, all the antibodies were purchased from cell signaling technology (CST, USA).

### Diabetic mouse skin wound model

Male C57BL/6J mice were given a high-sucrose and high-fat diet for 10 weeks, followed by intraperitoneal injection of streptozotocin (65 mg/kg) at 10 and 11 weeks. After 2 weeks, mice with random blood glucose higher than 16.7 mM/L were selected for further study. Obese or non-obese mice can be produced in this mouse model and mice were randomly divided into experimental group and control group, with each group intraperitoneal injection of 10% chloral hydrate (0.04 mL/10 g) and shaved the back hair. A full-thickness skin excision wounds with a diameter of 12 mm each were created. For EPCs-EXO treatment, the skin wounds were treated with EPCs-EXO (1mg/kg/wound) or PBS directly to the wound twice daily for two weeks. The area of the wound was measured by Image J software after the wound was photographed at days 0, 3, 5 and 7. For miRNA treatment, the pre-mRNA sequence of miR-182-5p was acquired through miRbase and constructed into the adenovirus vector of PDC-315, which was co-transfected with the helper plasmid pBHGloxdelE13cre in 293A cells for adenovirus packaging and recombinant adenovirus infectious titers have been measured by plaque assay. Mice were given a single intradermally injection into the wound edges of 10^8^ PFU immediately and 3, 5, and 7 days after wounding.

### siRNAs transfection

The small interfering RNA (siRNA) oligos against PPARG was commercially synthesized by Gene Pharma (Shanghai, China). Briefly, the HaCaTs (5x105 cells/well) were transfected by siRNA of PPARG or negative control with Lipofectamine RNAiMAX (Invitrogen, Carlsbad, CA, USA) according to the instructions of the manufacturer. After 4-6 h of incubation, the fresh medium was replaced and the cells were cultured for further experiments. Sequences for silencing PPARG: 5'- GCCAACATTTCCCTTCTTCCA-3'. The effect of RNA interference was double-checked by RT-PCR and Western blot analysis.

### Statistical analysis

All experiments were performed for three times, and the statistical analysis was performed using GraphPad Prism 8. Data are expressed as the mean ± SEM. Unpaired Student's t tests or two-way ANOVA were performed for comparisons between two groups or multiple comparisons, respectively. *p*<0.05 was considered statistically significant.

## Results

### Identification of exosomes derived from EPCs

**Figure 1:**
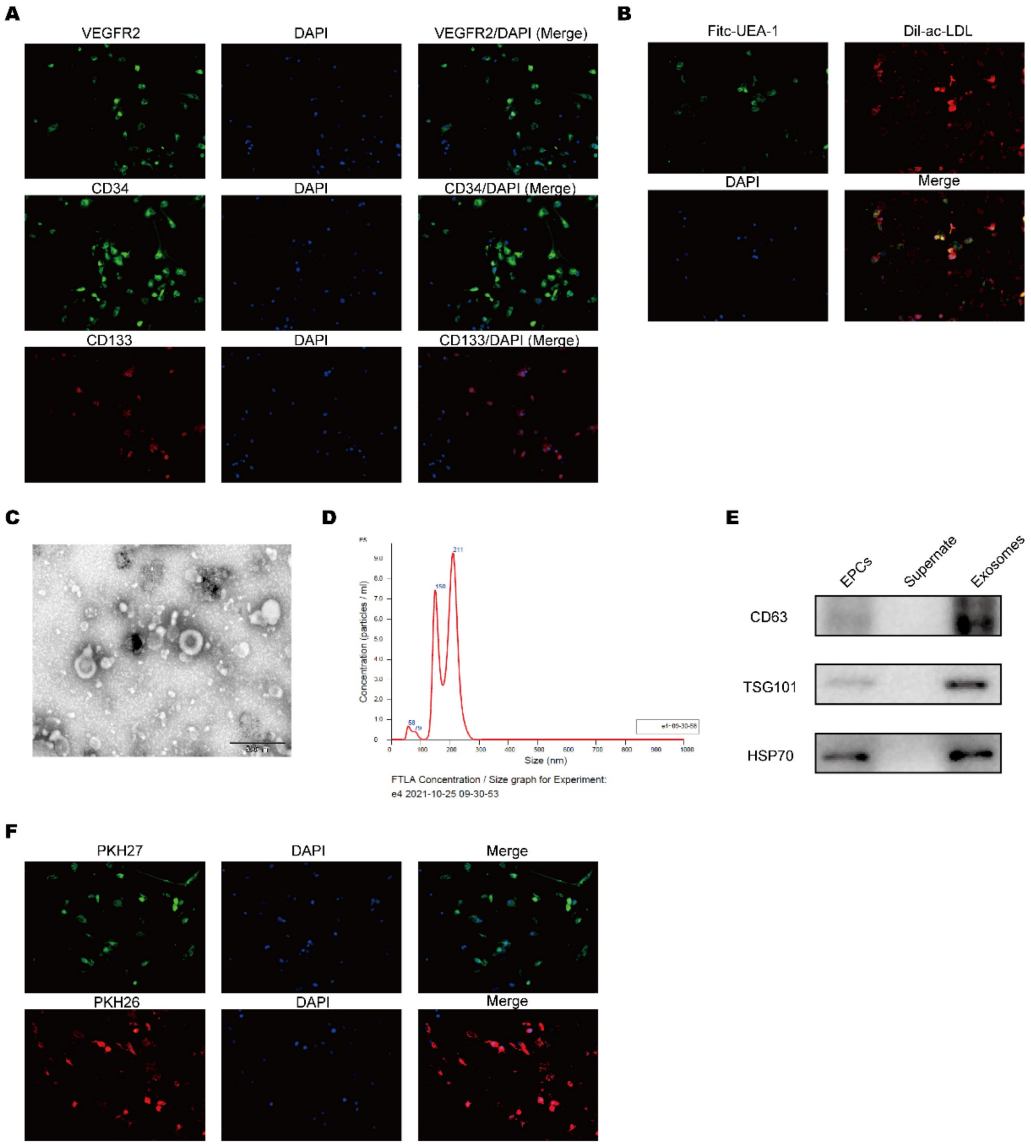
** Identification of exosomes derived from EPCs** (A) Immunofluorescence analysis of characteristic cell markers of EPCs. Green or red color represented measured markers (VEGFR2, CD34 and CD133, respectively) and blue color represent nucleus. (B) Immunofluorescence analysis of EPCs with fluorescence labeled Dil-ac-LDL and Fitc-UEA-1. (C) Representative micrographs showing exosome ultrastructure. Scale bar, 500 nm. (D) Exosome size distribution of EPC-EXOs. (E) Western blotting of EPC-EXOs surface marker protein expression levels. (F) Uptake of EPC-EXOs by HaCaTs was detected by PKH26 and PKH27. Scale bar, 20 μm.

EPCs separated from cord blood and the expression of the surface specific proteins CD34, CD133 and VEGFR2 on EPCs were detected by using immunofluorescence and these markers were strongly expressed (Figure 1A). To further identify the characteristics of EPCs, the cells were co-cultured with fluorescence labeled Dil-ac-LDL and Fitc-UEA-1 (Figure 1B). These data demonstrated that the cells were EPCs. After we purified EPCs-EXO, the morphological features were identified by electron microscope (Figure 1C). The size distribution analysis showed the diameter of EPCs-EXO ranged from 50 to 200 nm (Figure 1D). In addition, the exosomes specific marker proteins like CD63, TSG101 and HSP70 were highly expressed confirmed by Western blot (Figure 1E). Upon labeling them with PKH26 and PKH27, these EPCs-EXO could be readily picked up by HaCaT cells (Figure 1F). These data showed that EPCs-EXO are successfully isolated and readily taken up by HaCaT cells.

### Exosomes derived from EPCs enhance the proliferation, migration and inhibit apoptosis of HaCaT cells under HG condition

**Figure 2:**
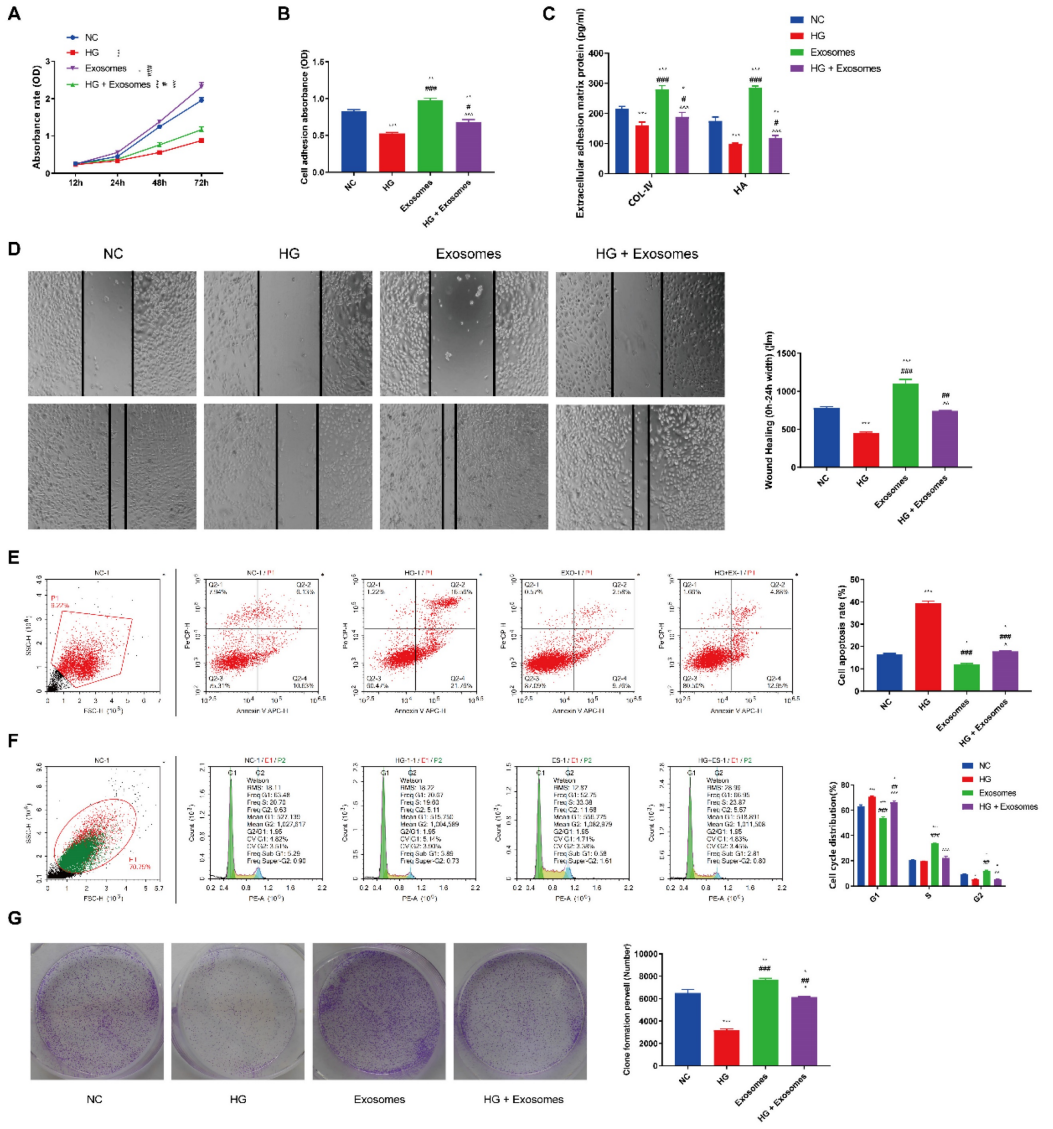
** Exosomes derived from EPCs enhance the proliferation, migration and inhibit apoptosis of HaCaT cells under HG condition** (A) CCK8 assay of HaCaTs viability after treated with EPC-EXOs under normal glucose (NG, 6 mM) or high glucose (HG, 33 mM) culture conditions. (B) Cell adhesion assay of HaCaTs after treated with EPC-EXOs under NG or HG culture conditions. (C) Extracellular adhesion matrix protein expression by ELISA of HaCaTs after treated with EPC-EXOs under NG or HG culture conditions. (D) Would scratch assay of HaCaTs after treated with EPC-EXOs under NG or HG culture conditions. (E) Flow cytometry of HaCaTs apoptosis after treated with EPC-EXOs under NG or HG culture conditions. (F) Flow cytometry of HaCaTs cell cycle after treated with EPC-EXOs under NG or HG culture conditions. (G) Clone formation assay of HaCaTs after treated with EPC-EXOs under NG or HG culture conditions. All experiments were repeated independently for at least three times. Statistical evaluation was performed using one-way ANOVA. Mean ± SEM. *represents *P* < 0.05 compared with NC group. *P < 0.05, **P < 0.01, ***P < 0.001; ^#^ represents *P* < 0.05 compared with HG group. ^#^*P* < 0.05, ^##^*P* < 0.01, ^###^*P* < 0.001; ^represents *P* < 0.05 compared with exosome group. ^*P* < 0.05, ^^*P* < 0.01, ^^^*P* < 0.001.

To test our hypothesis that EPCs-EXO could impact the biological phenotypes of HaCaT cells under diabetic condition, we conducted *in vitro* assays to explore the role of EPCs-EXO in HaCaT cells under HG environment. CCK8 assay results showed that HG culture conditions significantly reduced cell viability compared with NG culture conditions while EPCs-EXO treatment increased cell viability (Figure 2A). As shown in Figure 2B, adhered cells were quantitated by measuring absorbance at 540 nm, EPCs-EXO treatment promoted the adhesion ability which was inhibited under HG condition. In addition, cell adhesion molecules expression like collagen type IV and Hyaluronan (HA) were also upregulated after treatment with EPCs-EXO (Figure 2C). HaCaT cells migration, cell cycle and clone formation were all significantly enhanced while the apoptosis was inhibited after EPCs-EXO treatment under HG culture conditions (Figure 2D-2G). These results suggested that EPCs-EXO may play an important role in wound healing.

### Exosomes derived from EPCs promote wound healing in diabetic mice

**Figure 3:**
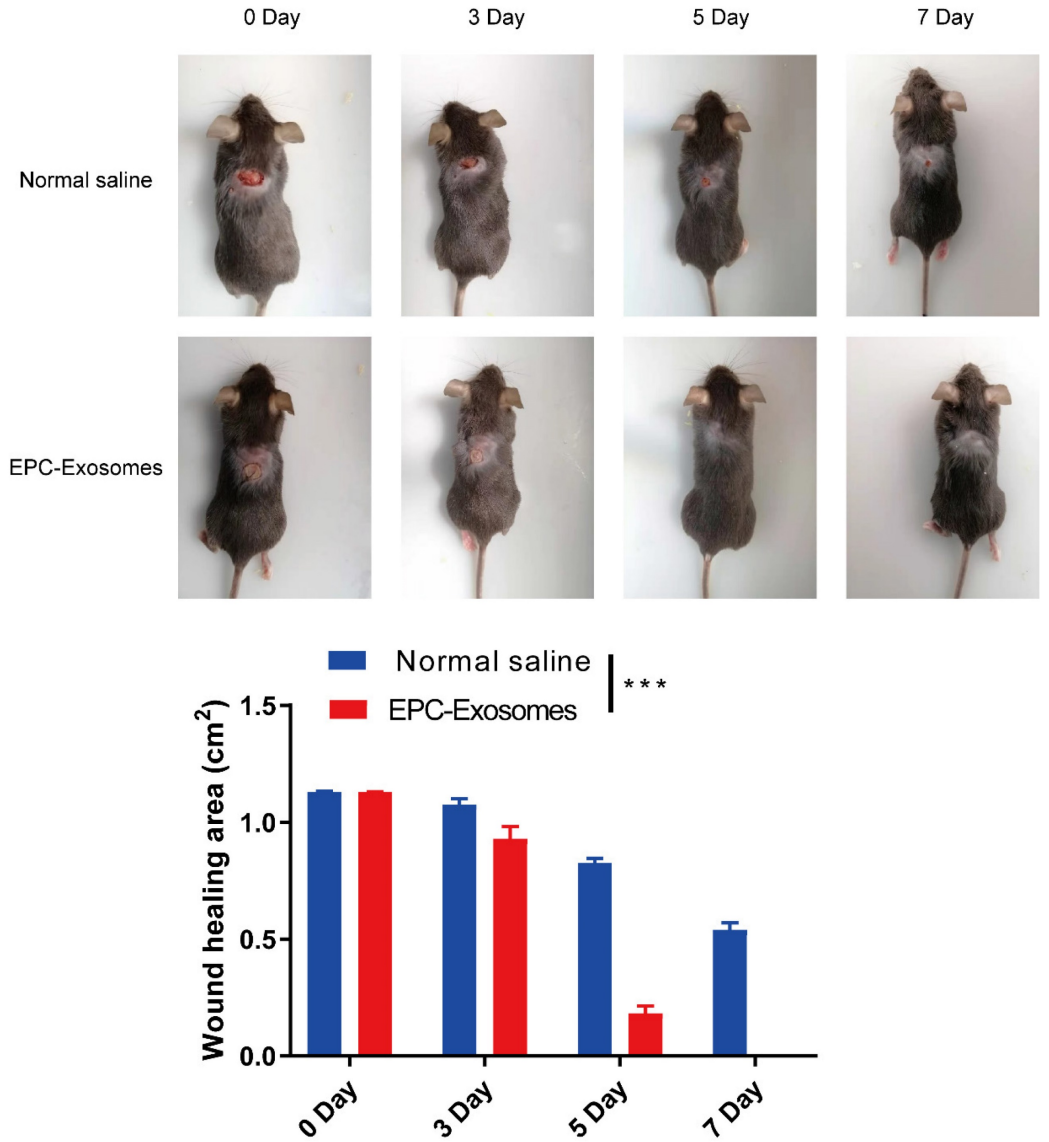
** Exosomes derived from EPCs promote wound healing in diabetic mice** Representative images and summary data showing skin wounds area in diabetic mice treated with PBS or EPC-EXOs. Values represent the mean ± SEM (n = 6). Statistical evaluation was performed using two-way ANOVA. ***P < 0.001.

To explore the potential of EPCs-EXO for clinical treatment, a full-thickness wound model at the dorsum of diabetic mice was established and the EPCs-EXO were used to treat the mice, PBS was used as a negative control. As shown in Figure 3, diabetic mice treated with EPCs-EXO showed accelerated wound closure compared with that in PBS-treated mice. These data indicated that EPCs-EXO significantly accelerated diabetic wound healing.

### Bioinformatics analyses of differentially expressed miRNAs

**Figure 4:**
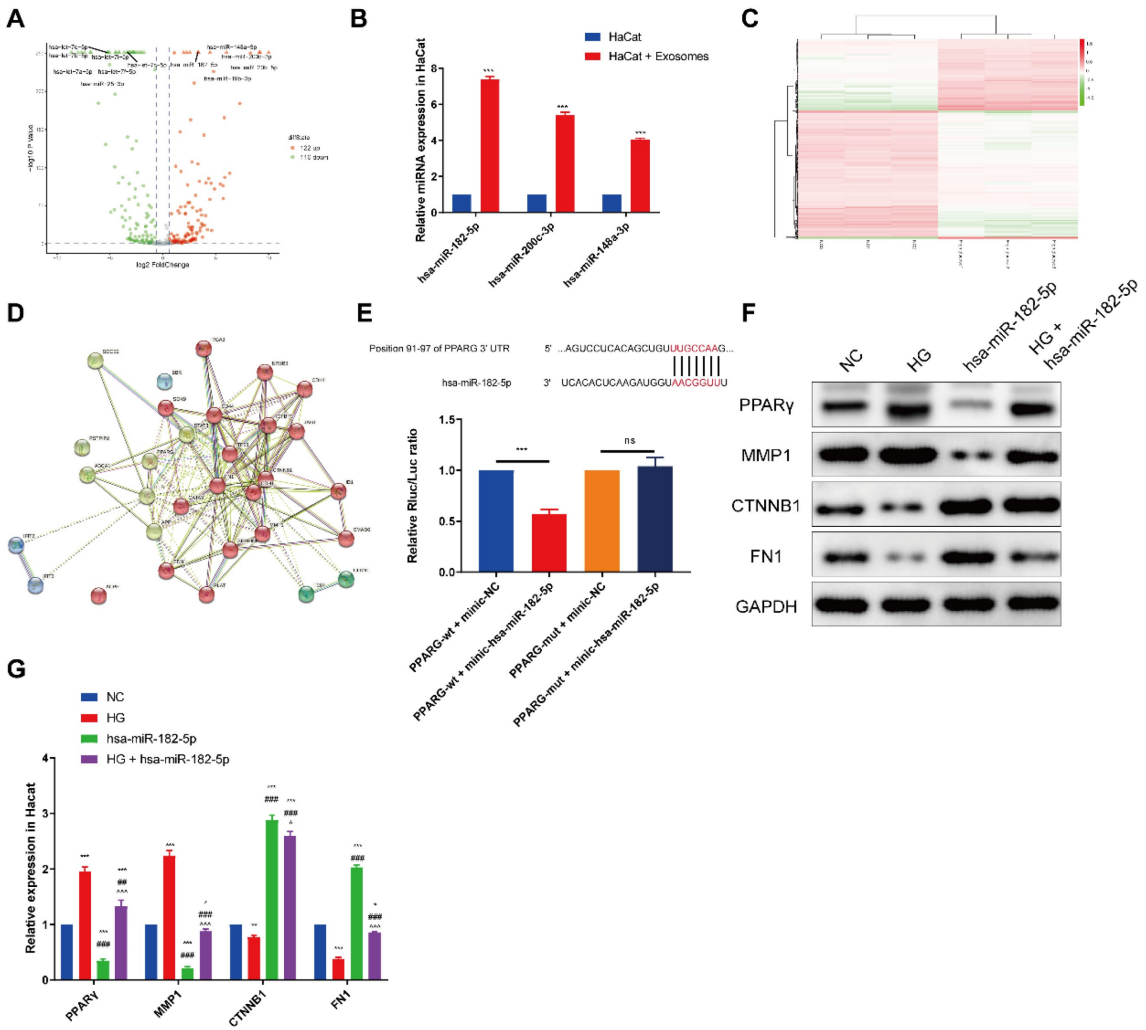
** Bioinformatics analyses of miRNAs in exosomes derived from EPCs** (A) Volcano plot of aberrantly expressed miRNAs in HaCaTs after treated with EPC-EXOs under HG culture conditions. (B) RT-PCR analysis of relative miR-182-5p, miR-200c-3p and miR-148a-3p levels in HaCaTs after treated with EPC-EXOs under HG culture conditions. Statistical evaluation was performed using two-way ANOVA. Mean ± SEM. ***P < 0.001. (C) Heatmap of differentially expressed genes in HaCaTs under NG and HG culture conditions. (D) Protein-protein interaction network of target genes among differentially expressed genes, top 20 KEGG and GO pathways. (E) Luciferase reporter assay demonstrated the association between miR-182-5p and PPARG. (F) Western blotting showed the expression of PPARG, MMP1, CTNNB1, FN1 and GAPDH in HaCaTs after treated with miR-182-5p under HG culture conditions. All experiments were repeated independently for at least three times. Statistical evaluation was performed using two-way ANOVA. Mean ± SEM. *represents *P* < 0.05 compared with NC group. *P < 0.05, **P < 0.01, ***P < 0.001; ^#^ represents *P* < 0.05 compared with HG group. ^##^*P* < 0.01, ^###^*P* < 0.001; ^represents *P* < 0.05 compared with miR-182-5p group. ^*P* < 0.05, ^^^*P* < 0.001.

To define the specific exosomal miRNA that participates in this progress, the expression profiles of miRNAs by sequencing analysis was assessed. A total of 232 aberrantly expressed miRNAs, among which 122 miRNAs were upregulated while 110 miRNAs were downregulated in EPCs-EXO (Figure 4A). As shown in Figure 4A, miR-182-5p, miR-148-3p, miR-200c-3p, miR-20b-5p and miR-19b-3p were high up-regulated in EPCs-EXO which was also validated by qRT-PCR in HaCaT cells treatment with EPCs-EXO for 48 h (Figure 4B). Moreover, miR-182-5p one of the most up-regulated miRNA in both EPCs-EXO and HaCaT cells treatment with EPCs-EXO, which indicating that miR-182-5p could be a potential target in promoting the wound healing of EPCs-EXO.

To investigate how miR-182-5p promotes the wound healing, the potential targets genes were predicted using microarray analysis in HaCaT cells treatment with EPCs-EXO under HG or NC environment. Heatmap showed the differentially expressed genes in HaCaTs treatment with EPCs-EXO under NG and HG culture conditions (Figure 4C). Protein-protein interaction network showed the potential target genes among differentially expressed genes, top 20 KEGG and GO pathways (Figure 4D). Our research was directed towards finding putative miR-182-5p targets combined with TargetScan and miRDB, which eventually led us to one target gene worth validation namely PPARG. Then the luciferase reporters (PPARG-WT and PPARG-mut) were constructed and transfected into the 293T cells. It was seen that overexpression of miR-182-5p significantly inhibited the luciferase activity of PPARG-WT compared with negative control, while had no effects on PPARG-mut cells (Figure 4E). Western blotting also showed that the expression of PPARG was inhibited after miR-182-5p overexpressed HaCaTs under HG condition (Figure 4F and 4G). As HaCaTs cell adhesion molecules expression was upregulated after treatment with EPCs-EXO, the expression of matrix metallopeptidase 1 (MMP1), fibronectin-1 (FN1) and catenin beta 1 (CTNNB1) was also evaluated. Our data showed that the expression of MMP1 was inhibited, while FN1 and CTNNB1 were upregulated after miR-182-5p overexpressed HaCaTs under HG condition (Figure 4F and 4G). Collectively, these results revealed that PPARG was a direct target of miR-182-5p and miR-182-5p treatment promotes the cell adhesion molecules expression.

### miR-182-5p/PPARG regulates HaCaTs functionality

**Figure 5:**
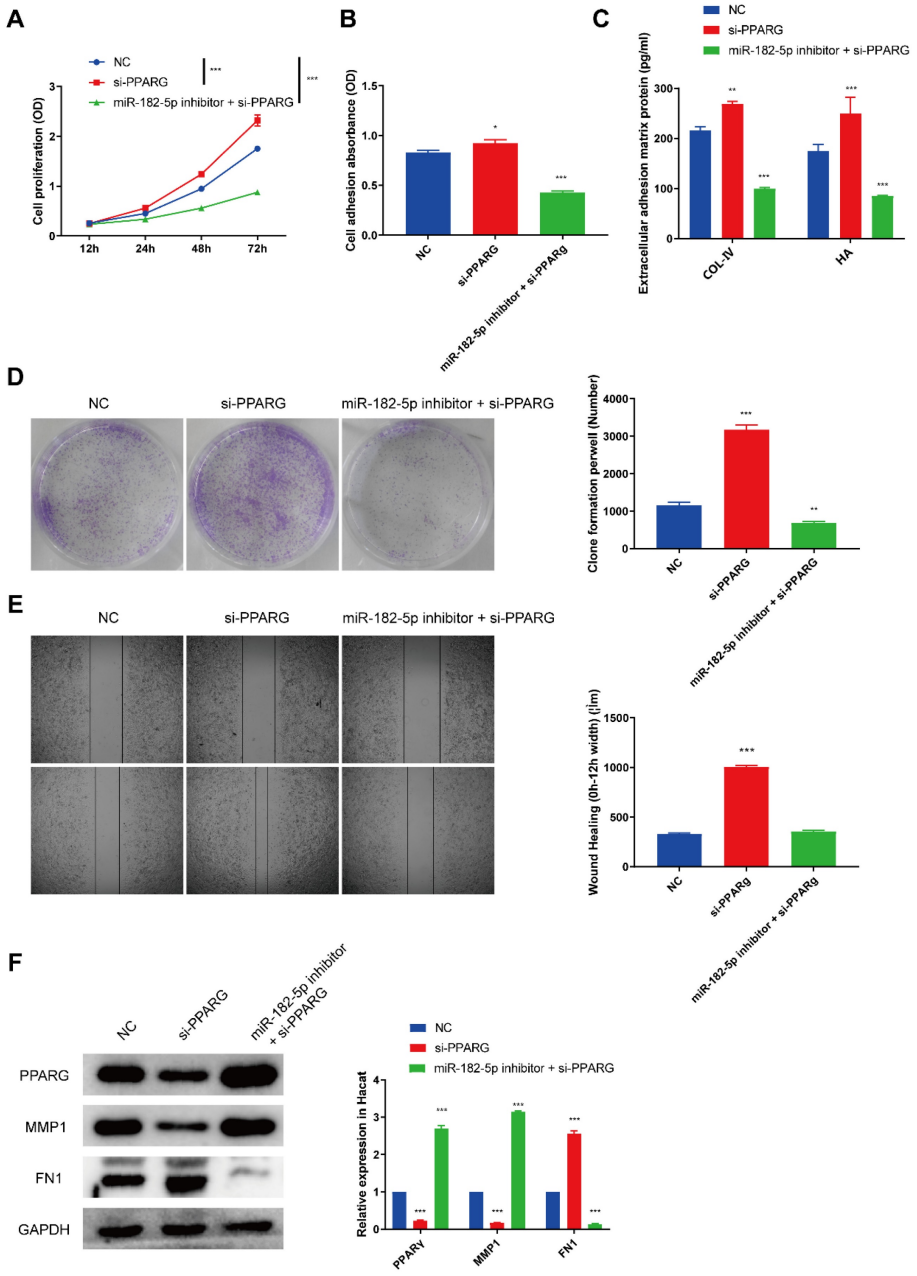
** miR-182-5p/PPARG regulates HaCaTs functionality** (A) CCK8 assay of HaCaTs viability after treated with si-PPARG or miR-182-5p inhibitor plus si-PPARG. (B) Cell adhesion assay of HaCaTs after treated with si-PPARG or miR-182-5p inhibitor plus si-PPARG. (C) Extracellular adhesion matrix protein expression by ELISA of HaCaTs after treated with si-PPARG or miR-182-5p inhibitor plus si-PPARG. (D) Clone formation assay of HaCaTs after treated with si- PPARG or miR-182-5p inhibitor plus si-PPARG. (E) Would scratch assay of HaCaTs after treated with si-PPARG or miR-182-5p inhibitor plus si-PPARG. (F) Western blotting showed the expression of PPARG, MMP1, FN1 and GAPDH in HaCaTs after treated with si-PPARG or miR-182-5p inhibitor plus si-PPARG. All experiments were repeated independently for at least three times. Statistical evaluation was performed using one-way ANOVA. Mean ± SEM. *represents *P* < 0.05 compared with NC group. *P < 0.05, **P < 0.01, ***P < 0.001.

As we found that PPARG was a direct target of miR-182-5p, we then tested the biological function of PPARG on HaCaTs via siRNA to inhibit the expression of PPARG. CCK8 cell proliferation assay (Figure 5A) was carried out and the result indicated that si-PPARG could exhibited a notable pro-proliferation effect and obviously counteracted miR-182-5p inhibitor suppressed HaCaTs proliferation. Cell adhesion assay (Figure 5B) and the expression of extracellular adhesion matrix protein (Figure 5C) also showed that si-PPARG could improve HaCaTs adhesion. Clone formation assay (Figure 5D) also showed that si-PPARG increased the proliferation of HaCaTs. We supposed that the knockdown of PPARG could promote the migration of HaCaTs and the subsequent *in vitro* wound scratch assay confirmed the hypothesis (Figure 5E). We also demonstrated that PPARG inhibition suppressed the expression of MMP1 and increased FN1 expression which abolished the effect of miR-182-5p inhibitor (Figure 5F). In summary, these data confirmed that miR-182-5p promoted HaCaTs functionality via targeting PPARG.

### miR-182-5p promotes wound healing in diabetic mice

**Figure 6:**
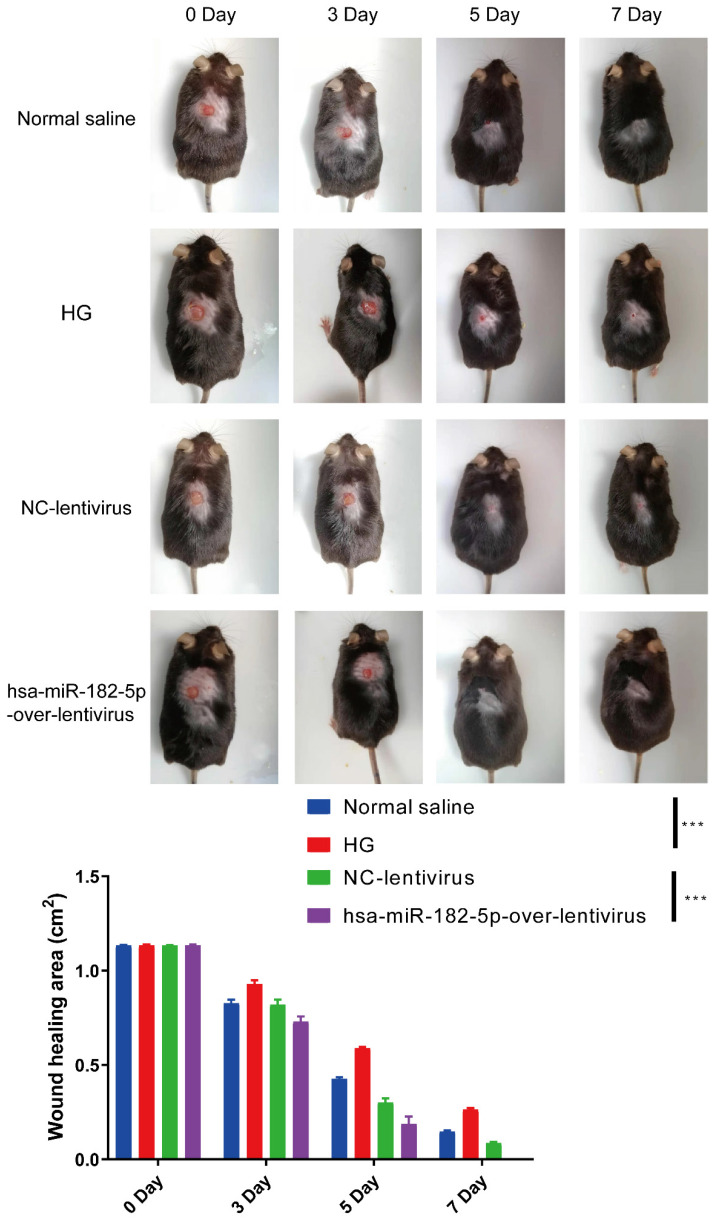
** miR-182-5p promotes wound healing in diabetic mice** Representative images and summary data showing skin wounds area in diabetic mice treated with negative control or miR-182-5p overexpression under HG condition. Values represent the mean ± SEM (n = 6). Statistical evaluation was performed using two-way ANOVA. ***P < 0.001.

In order to confirm the effect of miR-182-5p in wound healing, the *in vivo* diabetic wound was established and the miR-182-5p overexpression adenovirus was used to treat the mice. As shown in Figure 6, the healing rates of diabetic mice were significantly increased after treated with miR-182-5p overexpression adenovirus compared with negative control under HG condition. These data indicated that miR-182-5p promotes wound healing in diabetic mice and contributes to the regulation of EPCs-EXO.

## Discussion

The progression of diabetic wounds involves many pathophysiological processes, in which the function of epidermal cells is impaired, there is an urgent need to explore the mechanisms of re-epithelialization dysfunction for developing new pharmaceutic interventions^30^. Multiple researches have demonstrated that the treatment of stem cells and its derived exosomes is an effective way to treat diabetic foot wounds. Recent studies have found EPCs can effectively improve diabetic wound healing^31^. Our results found that EPCs-EXO can promote human immortalized epidermal cell line HaCaT proliferation, migration, adhesion and inhibit apoptosis under HG condition by miR-182-5p via inhibiting the expression of PPARG. In addition, EPCs-EXO also enhanced skin wound healing in diabetic mice via miR-182-5p. Our data justify the significance and the potential of EPCs-EXO in treating diabetic cutaneous wounds.

EPCs have gained surprising interest in stem cells and regenerative medicine research and improved clinical outcomes in patients with diabetic wound, cardiovascular disease or cancer due to their powerful paracrine effects in clinical practice. Extracellular vesicles (EVs) are lipid membrane-enclosed vesicular structures, which having important roles in cell-to-cell communication and both physiological and pathological effects 32. Functions of cell-derived EVs have also been reported in promoting proliferation or angiogenesis 33 and preventing inflammation 34 due to its releasing various active molecules. There are three main categories of EVs: apoptotic bodies, microvesicles and exosomes. Exosomes are the smallest of the vesicles and stem cell derived exosomes stimulate the proliferation, migration and angiogenesis of vascular endothelial cells to promote diabetic wound healing. Previous studies have shown that EPCs-EXO can regulate the function of endothelial cells and play an important role in the repair of damaged vascular endothelium. Zhang et al. had demonstrated that EPCs-EXO accelerate cutaneous wound healing by promoting angiogenesis through Erk1/2 signaling 35. Li et al. also showed that EPCs-EXO enhance the proliferation, migration and tube formation of vascular endothelial cells and increase the expression of angiogenesis-related molecules, indicating that EPCs-EXO facilitate wound healing by positively modulating vascular endothelial cells function 36. Our data also showed that EPCs-EXO promotes epidermal cell line HaCaTs proliferation, migration and adhesion *in vitro* and also accelerate the wound healing* in vivo*, which is also consistent with the previous studies.

Stem cell derived exosomes contain critical functional miRNAs that promote angiogenesis and transport them to target cells to improve function or activate endogenous repair mechanisms to treat diabetic wounds. miR-21 is highly expressed in exosomes derived from adipose mesenchymal stem cells, which significantly accelerates the process of wound healing and promotes the migration and proliferation of HaCaT cells, which may affect matrix metalloproteinase 2 and metalloproteinase tissue inhibitor 1 through the PI3K/AKT pathway 37. miR-126 promoted the proliferation, migration and angiogenesis of endothelial cells by activates Raf/ERK signaling in EPCs-EXO and down-regulated the expression of SPRED1^38^. Here in our study, miR-182-5p was selected from miRNA candidates in EPCs-EXO to accelerate diabetic wound healing. In addition, miR-182-5p was also significantly up-regulated after EPCs-EXO treatment under HG condition. However, there is no relative research about miR-182-5p and wound repair in diabetes. miR-182 has been demonstrated that promote epithelial-mesenchymal transition, invasion and migration and angiogenesis in both breast cancer 39 and HCC 40. Meanwhile, miR-182-5p could promote the proliferation, migration and angiogenesis of HUVECs 41, indicating the potential role of anti-angiogenic effect in endothelial cells of diabetes. In the current study, we demonstrated that miR-182-5p can positively regulate the diabetic wound healing by targeting PPARG.

PPARG was initially found to be critical for the differentiation and function of fat cells, but now it has also been found to play an important role in the cardiovascular system through promoting angiogenesis in vascular smooth muscle cells and vascular smooth muscle cells 42. Previous studies had shown PPARG activation improved the angiogenic potential, proliferation, and migration of EPCs which are impaired in type 2 diabetes 43. Previous studies had shown PPARG activation improved the angiogenic potential, proliferation, and migration of EPCs which are impaired in type 2 diabetes. PPARG can also forms a transcriptional complex with signal transducer and activator of transcription 5 (STAT5) to control EPC cell-cycle progression 44. In addition, agonist-dependent PPARG expression can inhibit EPC cell cycle progression, inhibit EPC proliferation, and promote impaired hematopoietic progenitor cell function 45. PPARg agonists, such as pioglitazone, have been shown to ameliorate the imbalance between endothelial damage and repair in diabetic patients 46. However, PPAR agonists have also been shown to impede hematopoietic progenitor cell function by inhibiting STAT5 gene expression 47. Physiological PPARG agonists, such as PA, can actually cause vascular injury in diabetic patients by impairing the bioavailability of EPC 48. Therefore, the study on analyzing the effect of PPARG activation on vascular cells is still controversial and further studies are needed. Here in our study, we validated that PPARG is a direct target of miR-182-5p through bioinformatics analysis, PCR analysis and luciferase reporter assay. What's more, the expression of PPARG was upregulated during HG condition while miR-182-5p downregulated the expression of PPARG. Silencing PPARG suppressed HaCaTs proliferation which abolished the effect of miR-182-5p inhibitor, indicating that miR-182-5p promoted HaCaTs functionality via targeting PPARG.

Diabetes wound healing is a dynamic process that overlaps proliferation, inflammation and coagulation, among which FN1 and MMP1 play a key role in wound healing. FN1 is a member of the glycoprotein family and involved in cell adhesion and migration processes in various cell types 49. MMPs play an indispensable role in wound healing via angiogenesis and cell migration during proliferation and remodeling. MMPs are expressed at low levels in normal tissues while upregulated in diabetes. Moreover, increased levels of MMP-1 have been identified in slow to heal wounds 50. Here in our study, we found that the expression of MMP1 was suppressed and the expression of FN1 was upregulated after miR-182-5p overexpression or PPARG knockdown, which indicating EPCs-EXO promote the proliferation, migration and adhesion in HaCaTs under HG condition via miR-182-5p/PPARG signaling pathway.

## Conclusions

In conclusion, our data showed that EPCs-EXO significantly improved endothelial cell proliferation, migration and enhanced the healing process of the diabetic wound under HG culture conditions via miR-182-5p/PPARG signaling pathway, indicating EPCs-EXO may be a novel potential therapeutic target for diabetic wound healing.

## Supplementary Material

Supplementary figures.
