# Supplementary material for: Endothelial progenitor cell derived exosomes mediated miR-182-5p delivery accelerate diabetic wound healing via down-regulating PPARG
Source: Int J Med Sci. 2023 Feb 13;20(4):468–81. doi: 10.7150/ijms.78790 (PMC10087624; doi:10.7150/ijms.78790)
Supplement: Supplementary file 1 — Supplementary figures. [file ijmsv20p0468s1.pdf]

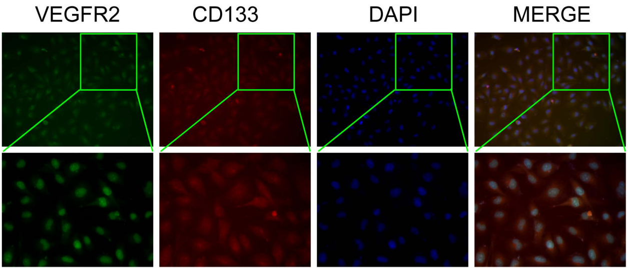
VEGFR2
CD133
DAPI
MERGE

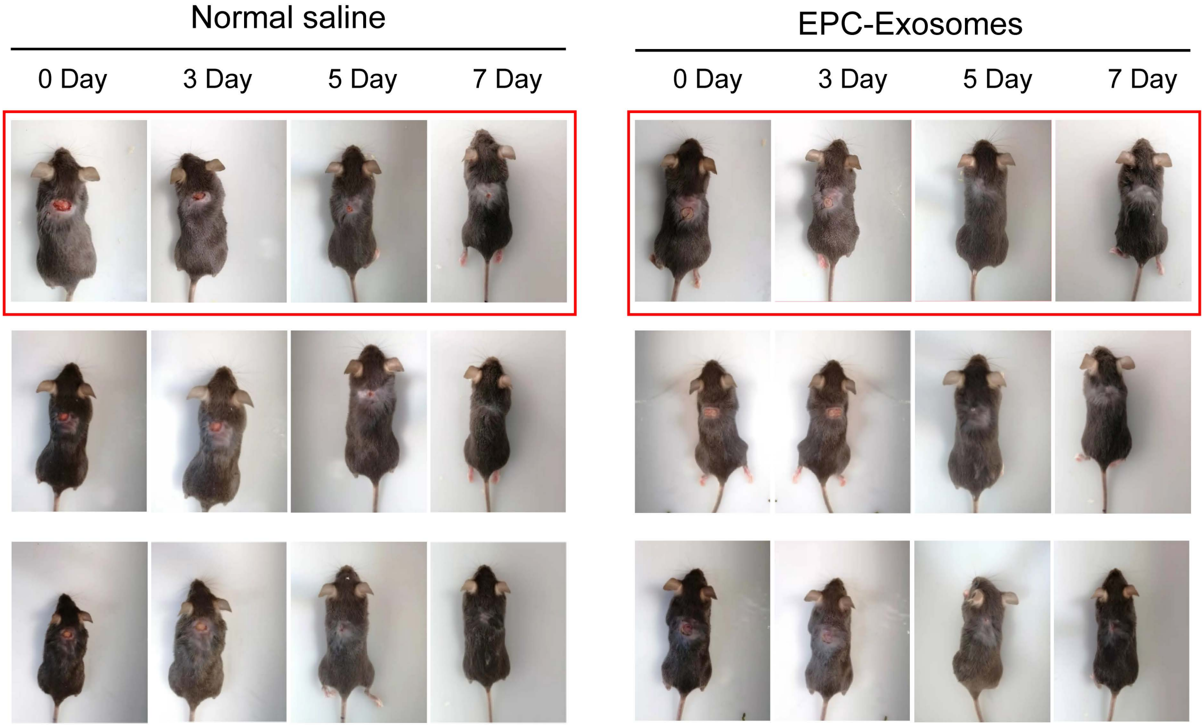

Normal saline
0 Day
3 Day
5 Day
7 Day
EPC-Exosomes
0 Day
3 Day
5 Day
7 Day

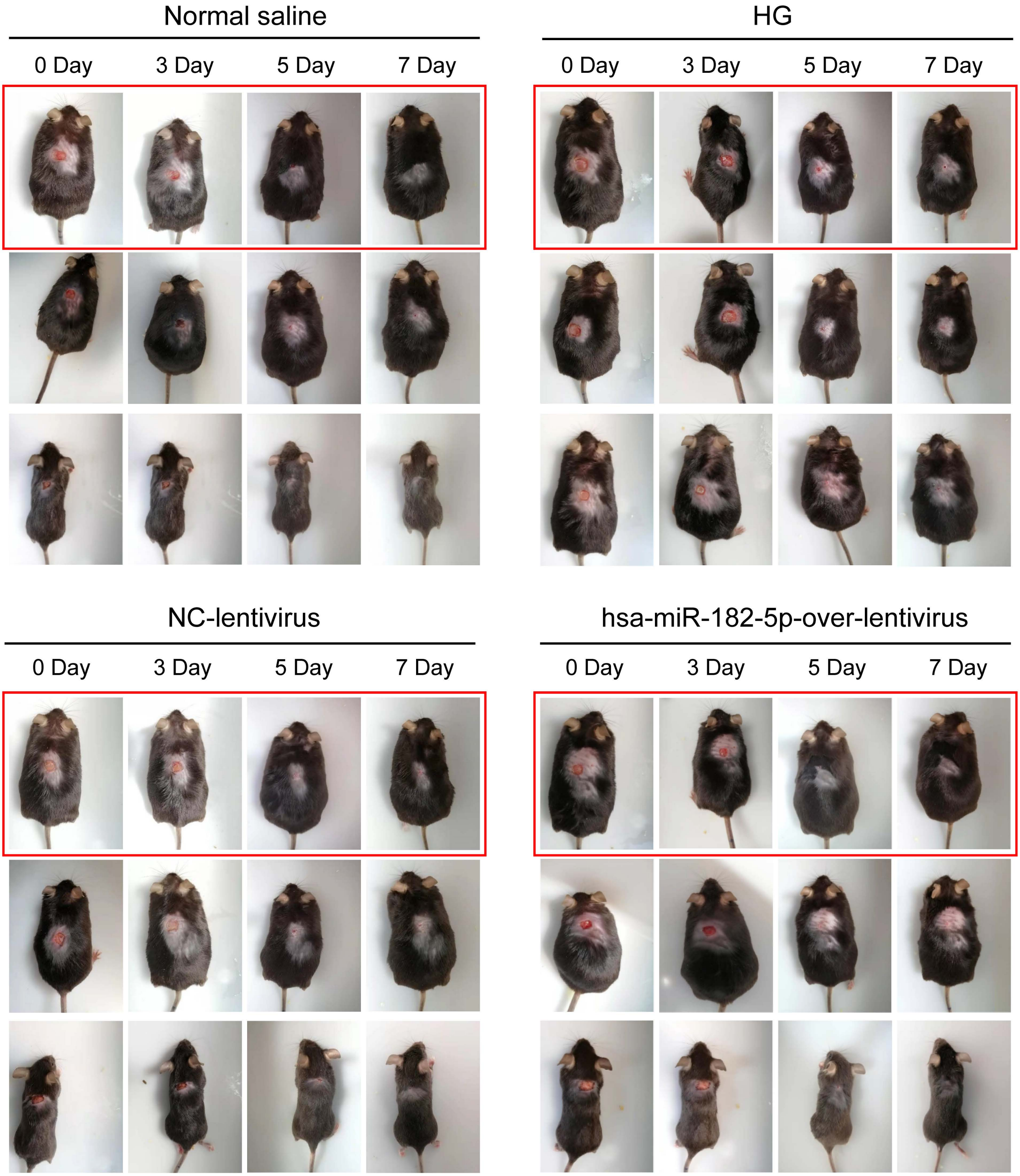
Normal saline
0 Day
3 Day
5 Day
7 Day
HG
0 Day
3 Day
5 Day
7 Day
NC-lentivirus
0 Day
3 Day
5 Day
7 Day
hsa-miR-182-5p-over-lentivirus
0 Day
3 Day
5 Day
7 Day
